# Inferior Vena Cava Tumor Thrombus Secondary to a Mixed Germ Cell Tumor: A Case Report of Multimodal Management With a Favorable Outcome

**DOI:** 10.7759/cureus.91383

**Published:** 2025-09-01

**Authors:** Abdalatiff K Bedaiwi, Rashad M Nassar, Abdullah M Alassaf, Abdulaziz A Albalawi, Hamad S Alakrash

**Affiliations:** 1 Urology, Prince Sultan Military Medical City, Riyadh, SAU; 2 Urology, King Khalid University Hospital, Riyadh, SAU

**Keywords:** bep chemotherapy, germ cell tumor, inferior vena cava thrombus, ivc filter, testicular cancer, testicular cancer survivor

## Abstract

Inferior vena cava (IVC) thrombus arising from testicular tumors is a rare clinical entity. Diagnostic imaging and subsequent pathological examination can help confirm a mixed germ cell tumor with an IVC thrombus. We present a case of a 19-year-old male with a right testicular tumor and IVC thrombus, successfully managed with a combination of a temporary IVC filter, anticoagulants, and systemic chemotherapy. The patient presented with a two-month history of right testicular swelling, significant weight loss, and other constitutional symptoms. He underwent right radical inguinal orchiectomy and received BEP (bleomycin, etoposide, and cisplatin) chemotherapy. Post-chemotherapy, the thrombus resolved completely, and the patient has remained disease-free for 24 months. This report highlights the presentation, diagnosis, treatment, and prognosis of metastatic testicular cancer with IVC tumor thrombus.

## Introduction

A testicular tumor refers to a growth on the testicles. These are germ cell tumors that can present as either benign (non-cancerous) or malignant (cancerous). Germ cell tumors are masses of tissue formed by immature cells that normally would have developed into mature eggs (in a female) or sperm (in a male). Of note, 90% of germ cell tumors are gonadal, i.e., they begin in the reproductive cells of the testes or ovaries. Testicular cancer is one of the most common malignancies in men aged 15 to 45 years. Its etiology is multifactorial, comprising various genetic and environmental factors.

An inferior vena cava (IVC) tumor thrombus is a serious condition where cancerous cells extend into the IVC, a large vein that carries blood from the lower body to the heart. Although common in renal cell carcinoma (RCC), IVC involvement originating from testicular cancer is rare. An IVC testicular tumor thrombus is a rare but serious complication of testicular cancer where a blood clot containing cancerous cells extends from the testicular veins into the IVC. Autopsy series have reported incidences of 2.6% to 11% in patients who died from testicular germ cell tumors [1-3]. Such involvement worsens prognosis, upstages disease, and complicates management [4]. We report a rare case of right testicular tumor with IVC thrombus, managed successfully with chemotherapy, anticoagulation, and IVC filter placement.

## Case presentation

**Figure 1:**
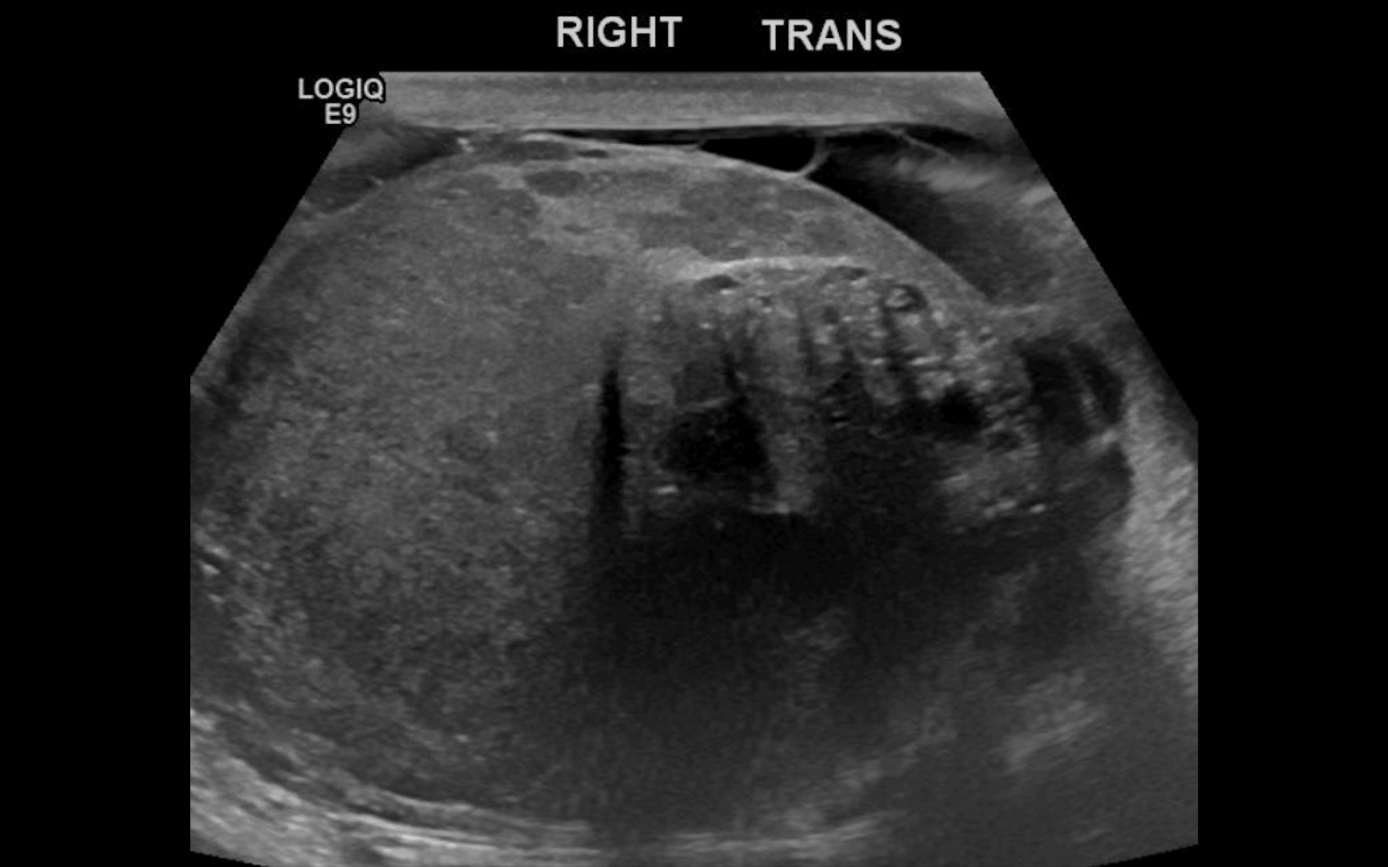
Right trans-scrotal ultrasound revealed a 4.7 x 5.3 x 8.4 cm heterogenous hypoechoic mass replacing the right testis with cystic spaces and macrocalcifications

A 19-year-old male with no prior medical history presented with a two-month history of right testicular swelling, nausea, vomiting, right lower abdominal pain, anorexia, and an 18 kg weight loss. Examination revealed a non-tender, firm right testicular mass. No palpable inguinal lymphadenopathy was noted. The patient had an Eastern Cooperative Oncology Group (ECOG) performance status of 1. Scrotal ultrasound showed a heterogeneous hypoechoic mass with cystic changes and macrocalcifications over the right testis (Figure 1).

Serum tumor markers revealed elevated lactate dehydrogenase (LDH) and mildly elevated alpha-fetoprotein (AFP), while beta-hCG was undetectable (Table 1).

**Table 1:** Preoperative laboratory results of tumor markers AFP: alpha-fetoprotein; LDH: lactate dehydrogenase; beta-HCG: beta human chorionic gonadotropin

Parameters	Patient values	Normal range
AFP, ng/ml	13	<10
LDH, U/L	1113	105-233
beta-HCG, mIU/mL	1	<2

**Figure 2:**
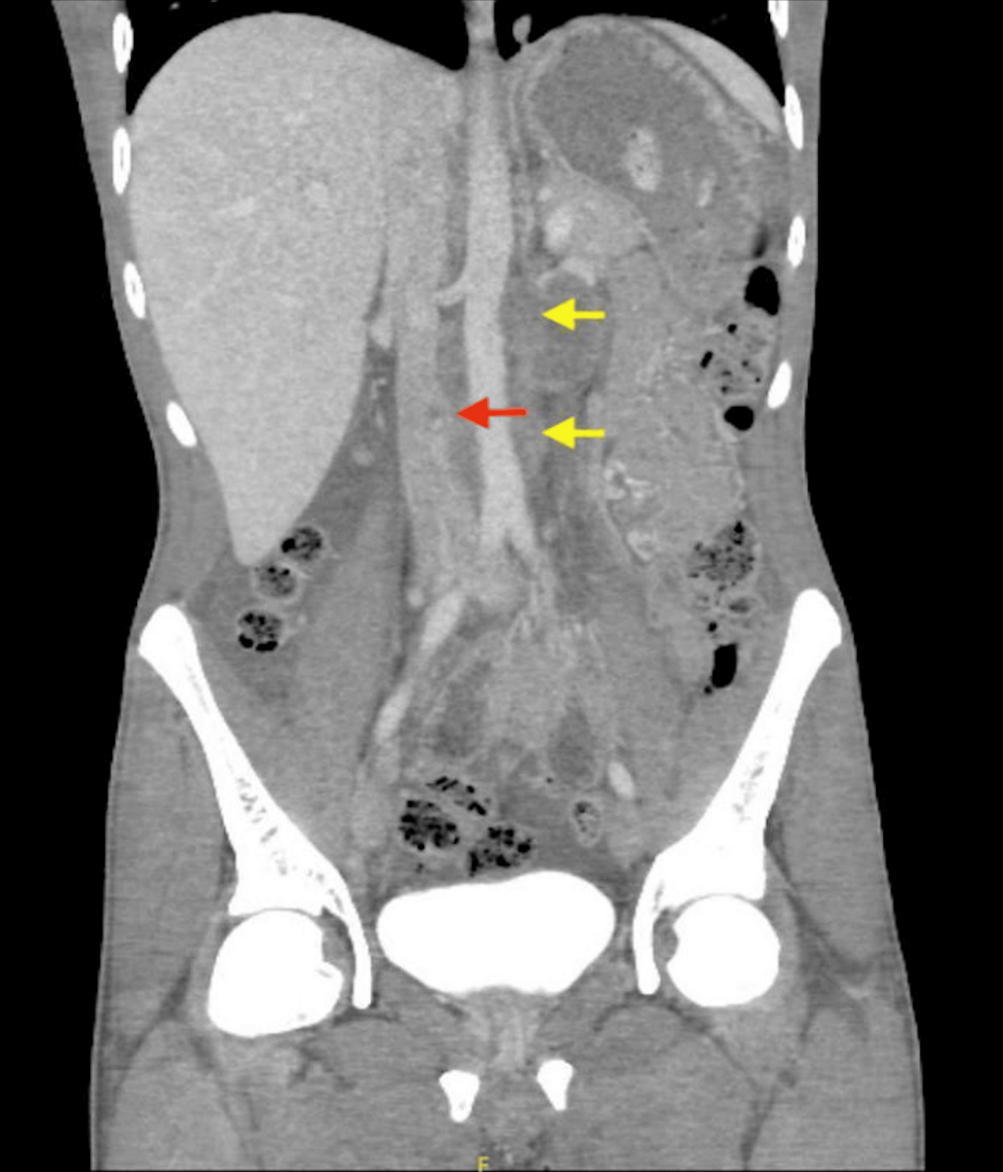
Coronal section of the CT scan of the abdomen The image demonstrated an IVC thrombus causing a filling defect measuring 8 mm, roughly 3.5 cm below the level of renal veins (indicated by the red arrow), with multiple para-aortic lymph node enlargement compressing the abdominal aorta (indicated by the yellow arrows) IVC: inferior vena cava; CT: computed tomography

CT of the chest, abdomen, and pelvis revealed a 27 mm para-aortic lymph node, enlarged interaortocaval, right iliac, and left supraclavicular lymph nodes, and multiple bilateral pulmonary metastases. An 8 mm IVC thrombus was identified 3.5 cm below the renal veins (Figure 2).

The patient underwent right radical inguinal orchiectomy. Histopathology revealed a mixed germ cell tumor (95% embryonal carcinoma, 5% teratoma) with lymphovascular invasion, rete testis, and epididymal involvement. Postoperatively, his AFP and LDH decreased (Table 2).

**Table 2:** Postoperative laboratory results of tumor markers AFP: alpha-fetoprotein; LDH: lactate dehydrogenase; beta-HCG: beta human chorionic gonadotropin

Parameters	Patient values	Normal range
AFP, ng/ml	1.1	<10
LDH, U/L	800	105-233
beta-HCG, mIU/mL	1	<2

The diagnosis was non-seminomatous germ cell tumor (NSGCT), stage pT2N2M1aS2, categorized as poor risk by International Germ Cell Cancer Collaborative Group (IGCCCG) criteria. A fluorine-18 fluorodeoxyglucose (F-FDG) positron emission tomography (PET) scan showed hypermetabolic bilateral pulmonary nodules and lymph nodes in the mediastinal, left supraclavicular, bilateral hilar regions, and bulky retroperitoneal regions extending to the right iliac group. Anticoagulation with enoxaparin was initiated, and an infrarenal IVC filter was placed via the right femoral vein.

Four cycles of BEP (bleomycin, etoposide, and cisplatin) chemotherapy were administered. Post-treatment PET showed complete metabolic resolution of metastases and normalization of tumor markers (Table 3).

**Table 3:** Post-chemotherapy laboratory results of tumor markers AFP: alpha-fetoprotein; LDH: lactate dehydrogenase; beta-HCG: beta human chorionic gonadotropin

Parameters	Patient values	Normal range
AFP, ng/ml	0.8	<10
LDH, U/L	151	105-233
beta-HCG, mIU/mL	1	<2

**Figure 3:**
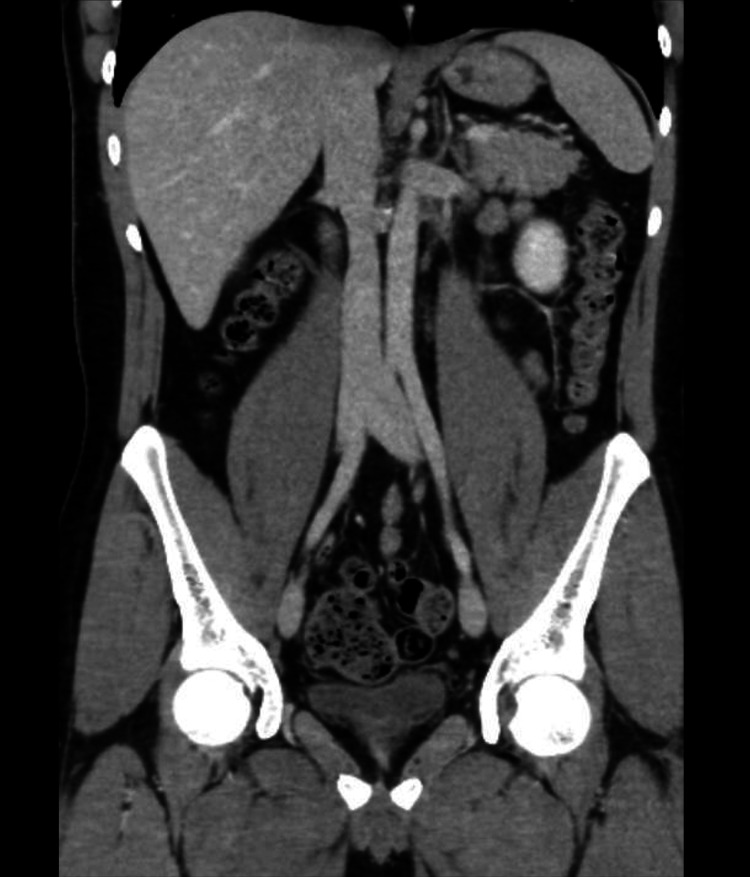
Coronal section CT scan of the abdomen after four cycles of chemotherapy The image demonstrated a patent IVC with no evidence of thrombus, along with resolution of para-aortic lymph node enlargement IVC: inferior vena cava; CT: computed tomography

The follow-up CT confirmed IVC patency without thrombus (Figure 3). The IVC filter was removed, and the patient remains disease-free at 24 months.

## Discussion

IVC tumor thrombus in testicular malignancies occurs via direct extension of the right spermatic vein in the case of right-sided testicular tumors or through the left renal vein in the case of left-sided testicular tumors or by lymphatic-venous shunting from retroperitoneal metastases [1]. IVC involvement can be either asymptomatic, as in the patient described in this case report, or clinically evident, related to IVC syndrome secondary to IVC obstruction, including hypotension, tachycardia, lower extremities edema, and varicocele, and in some instances, present with pulmonary embolism or sudden death secondary to distal embolization of the tumoral thrombus [4].

In the current case report, the treatment strategy for the IVC tumoral thrombus following radical orchidectomy aimed to achieve full remission of the IVC tumor thrombus through systemic chemotherapy, while also preventing pulmonary embolism through prophylactic anticoagulation and temporary IVC filter placement. This approach was employed by Masui et al., who reported complete remission of the IVC thrombus along with successful prevention of pulmonary embolism with no IVC filter-related complications [5]. Other case studies by Desaud et al. and Geffen et al. also reported complete regression of IVC tumor thrombus after four cycles of the BEP regimen, without the need for vena cava resection or thrombectomy [1,6].

Donohue et al. investigated the indications in 40 patients who underwent inferior vena caval resection, and two patients who underwent intraluminal tumor thrombectomy during retroperitoneal lymph node dissection for bulky abdominal metastatic testicular cancer. They reported that the objectives of such procedures were either to ensure complete tumor remission or the resection of a nonfunctional vena cava occluded by scar tissue or intraluminal thrombus [7]. Urgent thrombectomy was performed by O’Brien and Lynch, and Savarese et al. As the two case studies reported, the IVC tumor thrombus originating from testicular malignancy extended into the right atrium, increasing the risk of pulmonary embolism. Urgent thrombectomy was performed before chemotherapy, using cardiopulmonary bypass with hypothermia and cardiac arrest to obtain distal control during surgery [8,9].

Several case studies have investigated the histopathological composition of intraluminal tumoral IVC thrombus originating from testicular germ cell tumors. O’Brien and Lynch reported the histopathological findings of the IVC thrombus after thrombectomy, which was done before chemotherapy; they revealed an organized clot with foci of poorly differentiated seminoma compatible with the histopathology of pure seminoma with blastic giant cells of the primary testicular tumor [8]. Masui et al. performed post-systemic chemotherapy histopathological examination of the IVC thrombus captured during IVC filter retrieval in a patient with embryonal carcinoma of the testis; they reported necrotic cells with no viable tumor tissue [5].

## Conclusions

This report highlights a rare but serious case of IVC thrombus secondary to a mixed germ cell testicular tumor, along with demonstrating its diagnostic and therapeutic challenges. Utilizing a combination of radiological approaches, including CT and FDG PET scans, contributed to the detection of IVC tumor thrombus. Case outcomes support the evidence reported by previous case studies about the efficacy of cisplatin-based chemotherapy in achieving thrombus resolution and metastatic disease remission, along with the successful prevention of pulmonary embolism by temporary IVC filter placement and anticoagulation therapy. The findings also endorse the selective use of temporary IVC filters in testicular cancer patients with tumor thrombus, preventing embolic complications without necessitating major vascular surgery.
